# Our Philadelphia Letter

**Published:** 1886-03

**Authors:** Harry Huzza

**Affiliations:** Philadelphia


### Correspondence.

### OUR PHILADELPHIA LETTER.

THE RECENT COLD WEATHER—DR. THOMAS A. EMMETT'S VISIT TO JEFFERSON COLLEGE—HIS OPERATIONS BEFORE THE CLASS—THE CLINICS AT THE HOSPITALS—THE COUNTY MEDICAL SOCIETY, ETC.

*Editors Atlanta Medical and Surgical Journal:*

I suppose you have at last thawed out from the "blizzard," which I understand turned the Sunny South into a new Arctic region. We have been snowed in here since Christmas, but with skating and sleighing there was no lack of enjoyment in spite of the cold weather.

On January 12th, Dr. Thomas A. Emmett, of New York, held the Woman's Clinic at the Jefferson College Hospital. He operated two cases of *procidentia uteri*, and one of lacerated cervix, explaining his method of procedure and demonstrating the reasons for it. There were over seven hundred students present in the amphitheater to witness the operations. Dr. Emmett is a graduate of Jefferson, yet strangely enough this was the first time he had visited his *alma mater* since graduating thirty-four years ago.

In the surgical clinic Dr. Allis exhibited a simple means of distinguishing feigned from true fixations of the hip-joint. The apparatus consists of a rubber bag or a bladder filled with water and connected with a long glass tube. The patient being on his back, this bag is placed under the lumbar vertebræ, and the tube held upright. The limb is then elevated. If fixation exists the liquid will be forced up through the tube and even run out at the top. But if there be motion in the joint no effect will be produced on the liquid in the bag.

In the medical clinic was lately presented a case which interested me from its cause. The patient was a woman, aged 35, who complained of excessive flatulency and belching of gas. Had exhausted all the usual remedies; had simplified her diet in trying to cure herself, and cut off various articles of aliment, until she was now living almost entirely on oatmeal. But, in spite of this plain food, was losing flesh and growing steadily worse. Diagnosed as flatulent dyspepsia with sour gastric catarrh. The oatmeal being starchy, and in addition sweetened, thus becoming also saccharine, found a ready ferment in the catarrhal mucus, breaking up into acetic acid and carbonic acid gas. The treatment consisted essentially in regulating the diet; should eat underdone meat and easily digested animal food; must have no starchy or saccharine matters at all, and only such vegetables as contained little or no starch, tomatoes, spinach and celery. To prevent the fermentation in the stomach was given—

| | | |
|---|---|---|
| ℞. | Creasoti . . . . . . | gtt. i |
| | Bismuthi Sub. Nit. . . . . . | gr. xv |
| | Glycerinæ . . . . . . | ℥ ss |

To be taken in mint water three times a day. The next week the patient returned and reported herself cured.

There was also shown the case of a woman suffering from Bright's disease. She was in the pre-albuminuric stage, characterized by the passage in the twenty-four hours of large quantities of pale urine, but containing no albumen. There was tumultuous action of the heart and breathlessness on exertion. Had formerly slight swelling of legs, but now no dropsy. Arteries rigid and of high tension, showing arterial changes. No œdema of brain, but increase of fluid in peri-vascular lymph-spaces as evinced by constant headache.

The treatment consisted, first, in careful regulation of the diet. Was allowed milk and meat-broths, a few vegetables of succulent variety, some fruits if desired, but very little solid food. Cases of Bright's disease require two indications to be met by remedies : first, it is necessary to relax the high vascular tension, and thus relieve and benefit the system. For this purpose was given a one per cent. solution of nitro-glycerine, one drop of nitro-glycerine to one hundred drops of alcohol. Begin with one minim of this solution three times a day, and daily add a minim till the constitutional effects are produced as shown by frontal headache, flushing of the face and increased action of heart. In the second place, it is required to remedy the overgrowth of the kidney and act locally there. Was given chloride of gold and sodium 1-20 gr. in pill form, three times a day, gradually increased to 1-10 gr.

Perhaps it might be of interest to mention a convenient substitute for Fehling's solution in testing for sugar in the urine. The ordinary solutions deteriorate on keeping and are liable to throw down the sub-oxide of copper themselves if they have not been freshly prepared. Prof. Holland, in his lectures on chemistry, gave the following test fluid which is very efficient, is easily prepared and is not spoiled by keeping :

GLYCERINE CUPRIC SOLUTION.

| | |
|---|---|
| Cupric Sulphate, . . . . . . . . . . . . . | ʒ i. |
| Glycerine, . . . . . . . . . . . . . . . | f. ℥ i. |

To make the test add five drops of this solution to one drachm of *liquor potassæ* in a test-tube. Boil a few moments to test the purity of the fluid; should it remain clear, then add a few drops of urine. If glucose be present in quantity there is at once thrown down a red precipitate just as in the ordinary Fehling's test.

To detect minute amounts of sugar, not shown by above procedure, after making the test as above, add half a drachm of urine; boil and set aside. If sugar be present even in very minute quantity, the liquid as it cools will turn to an olive green color and become turbid.

At the last meeting of the County Medical Society, Dr. J. F. Edwards read a paper on "The Suppression of the Illegal Practice of Medicine in Philadelphia." He discussed the present registration law, showed its inefficiency, yet said it would do much good if properly enforced. In speaking of the qualifications upon which some of these pretended doctors based their claims, he told of one man who, originally a farmer, began to practice because he married the widow of a physician. Another quack had made affidavit that he had been in practice ten years, four of which it was found were spent in the penitentiary. There was quite an animated discussion of Dr. Edwards' paper by the members. The committee who have the matter in charge were assured of the hearty moral and financial support of the Society. The work of prosecuting these illegal practitioners will be pushed vigorously to the undoubted benefit of Philadelphia and its citizens. Truly yours,

Philadelphia, February 10, 1886. HARRY HUZZA.

